# Retroperitoneal lymph node dissection for residual masses after chemotherapy in nonseminomatous germ cell testicular tumor

**DOI:** 10.1186/1477-7819-8-97

**Published:** 2010-11-09

**Authors:** Murilo A Luz, Ahmed F Kotb, Saad Aldousari, Fadi Brimo, Simon Tanguay, Wassim Kassouf, Armen G Aprikian

**Affiliations:** 1 Division of Urology, Department of Surgery, McGill University, Montreal, QC, Canada; 2 Department of Pathology, McGill University, Montreal, QC, Canada

## Abstract

**Background:**

Retroperitoneal lymph node dissection has been advocated for the management of post-chemotherapy (PC-RPLND) residual masses of non-seminomatous germ cell tumors of the testis (NSGCT). There remains some debate as to the clinical benefit and associated morbidity. Our objective was to report our experience with PC-RPLND in NSGCT.

**Methods:**

We have reviewed the clinical, pathologic and surgical parameters associated with PC-RPLND in a single institution. Between 1994 and 2008, three surgeons operated 73 patients with residual masses after cisplatin-based chemotherapy for a metastatic testicular cancer. Patients needed to have normal postchemotherapy serum tumor markers, no prior surgical attempts to resect retroperitoneal masses and resectable retroperitoneal tumor mass at surgery to be included in this analysis

**Results:**

Mean age was 30.4 years old. Fifty-three percent had mixed germ cell tumors. The mean size of retroperitoneal metastasis was 6.3 and 4.0 cm, before and post-chemotherapy, respectively. In 56% of patients, the surgeon was able to perform a nerve sparing procedure. The overall complication rate was 27.4% and no patient died due to surgical complications. The pathologic review showed presence of fibrosis/necrosis, teratoma and viable tumor (non-teratoma) in 27 (37.0%), 30 (41.1%) and 16 (21.9%) patients, respectively. The subgroups presenting fibrosis and large tumors were more likely to have a surgical complication and had less nerve sparing procedures.

**Conclusion:**

PC-RPLND is a relatively safe procedure. The presence of fibrosis and large residual masses are associated with surgical complications and non-nerve-sparing procedure.

## Background

In accordance with the last report of The Public Health Agency of Canada (PHAC), the incidence of testicular cancer in Canada is rising and is the most common cancer in young men. The two main histologic subgroups occur with similar frequencies: 54% are seminoma and 41% non-seminoma germ cell tumors; 5% are other types[1].

Testicular cancer has become the model for a curable neoplasm. In treatment of nonseminomatous germ cell testicular tumors (NSGCTT), there have been great improvements in the last 25 years. Cure rates for clinical stage I and low-volume stage II testis tumor patients approach 100%; selecting the best initial modality of treatment and integration of surgery and chemotherapy is critical to optimizing cure and minimizing morbidity[2,3]. Furthermore, stage IIb and III metastatic NSGCT have very high cure rates owing to improvements in multi-drug chemotherapy protocols based on cisplatin. Nearly 80% of the patients presenting with retroperitoneal residual masses as the only site of metastasis after cisplatin-based chemotherapy can be cured by post-chemotherapy retroperitoneal lymphadenectomy (PC-RPLND). Of the patients requiring resection of residual disease after primary chemotherapy, approximately 90% will have either necrosis or teratoma in their resected specimens. This number decreases to 50% in patients undergoing resection after salvage chemotherapy[3,4]

Currently, RPLND of residual masses after cisplatin-based chemotherapy is a widely accepted procedure. There are some efforts to limit the extent of surgical resection boundaries to reduce complications[4]. In 40-50% of patients undergoing postchemotherapy resection of residual disease and bilateral RPLND, the histological diagnosis of the surgical specimen will be necrosis[5]. Thus, in a substantial proportion of patients, adjunctive surgery offers no additional therapeutic benefit. In an effort to select the patients who do not need surgery, some investigators have advocated the use of various radiographic and histological parameters. However despite these efforts, the risk of omitting surgery in a patient who harbors viable cancer or teratoma appears to be 20%[6,7].

Since it appears difficult to predict which patients require PC-RPLND for residual masses that harbor viable cancer or teratoma, there remains significant concern regarding surgical complications. We report our experience with PC-RPLND for residual disease and examine the complications as well as histologic and clinical outcomes.

## Methods

Between 1994 and 2008, three surgeons performed 81 RPLND for a residual mass(es) after cisplatin-based chemotherapy for clinical stages II or III testicular NSGCT. All patients were operated at the Montreal General Hospital (McGill University Health Center). The inclusion criteria were: nonseminomatous tumors, normal postchemotherapy serum alpha-fetoprotein and human chorionic gonadotrophin levels and no prior surgical attempts to resect retroperitoneal tumours. Exclusion criteria were: incomplete data, inadequate follow-up and surgical treatment performed in another hospital. Patients who underwent primary RPLND or radiotherapy were also excluded. PC-RPLND consisted of a full bilateral template limited by the renal vessels, the ureters and the bifurcation of common iliac vessels. Complications were reported according to the relatively new classification of surgical complications proposed by Clavien[8], using a scale from I to V. Statistical analysis was done using SPSS version 11.1 for descriptive analysis, comparing means of subgroups (independent t-tests) and comparing groups to surgical complications and presence of viable cancer (Chi-square test, Fisher's exact test). Independent predictors of complications and/or presence of viable disease were analyzed (age, laterality, comorbidities, initial histology, tumor markers, risk, initial tumor size, initial and residual mass size, surgical technique, operative time) The disease free survival rate (calculated from the time of retroperitoneal surgery until the first clinical evidence of recurrence) was calculated using a Kaplan-Meier curve and comparisons by Log Rank test.

## Results

Of the 81 cases, 73 fulfilled the inclusion criteria. The mean age was 30.6 years ranging from 16 to 58. Only 6 patients (8.2%) had some preoperative comorbidity including sarcoidosis (1), hypertension (1), chronic renal failure (1), epilepsy (1), atrial fibrillation (1) and duodenal ulcer (1). The patient with chronic renal failure underwent a kidney transplantation before the diagnosis of testis cancer. Chryptorchidism was present in 8 (11%) patients, with two being bilateral.

The primary tumor was located on the right side in 34 (46.6%) cases. The most prevalent histology of the primary tumor was mixed germ cell tumor, present in 50 cases (60.8%). Thirty-one patients (42.5%) presented some component of teratoma. Two cases showed burned-out tumours. The testis tumor characteristics are summarized in Table 1.

**Table 1:** Clinical and pathological features of testis tumors

Characteristic		n	(%)
Size (mean, cm)		3.5 (±2.0)	
Laterality	Left	34	(46.6)
	Right	37	(50.7)
Clinical Stage	IIA	9	(12.3)
	IIB	28	(38.4)
	IIC	22	(30.1)
	III	14	(19.2)
Histology	MGCT	50	(68.5)
	EC	13	(17.8)
	Teratoma	7	(9.6)
	Others	3	(4.2)
Tumor risk	Good	56	(76.7)
	Intermediate	10	(13.7)
	Poor	7	(9.6)
LV invasion		24	(32.9)
CIS		9	(12.3)

MGCT = mixed germ cell tumor, EC = Embryonal carcinoma, LV = lymphvascular, Ca = carcinoma in situ

**Figure 1:**
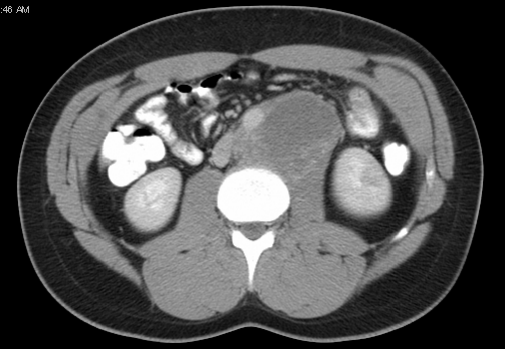
**Large post-chemotherapy residual paraortic mass (teratoma)**.

The mean size of retroperitoneal metastasis was 6.3 cm before chemotherapy and 4.0 cm after systemic treatment (p < 0.001). The most common sites of retroperitoneal metastasis was paraortic (Figure 1), paracaval and interaortocaval in 33 (45.2%), 19 (26.0%) and 14 (19.2%) patients, respectively.

All patients were submitted to cisplatin-based chemotherapy regimens. The most common was BEP (bleomycin, etoposide and cisplatin) for 2 to 4 cycles in 57 patients (78.1%). EP (etoposide and cisplatin), VIP (vinblastin, ifosfamide and cisplatin) or a combination of regimens were used in 3 (4.1%), 4 (5.5%) and 9 (12.3%), respectively. Additional treatments to normalize the markers were necessary in 7 cases (9.6%) and two patients were submitted to bone marrow transplantation to rescue high dose chemotherapy treatment. The characteristics of post-chemotherapy retroperitoneal disease are described in Table 2.

**Table 2:** Clinical characteristics of retroperitoneal metastasis

Characteristic		n	(%)
Size	Before CT	6.3 (±3.9)	p < 0.001
	After CT	4.0 (±2.6)	
Main site	Paraortic	33	(45.2)
	Paracaval	19	(26.0)
	Interaortocaval	14	(19.2)
	Precaval	3	(4.1)
	Retrocural	3	(4.1)
	Iliac	1	(1.4)

CT = chemotherapy treatment

The surgical approach was abdominal in 70 (95.9%) cases and thoracoabdominal in 3 cases. A complete bilateral retroperitoneal dissection was performed in 67 (92.0%) patients. In six (8.0%) patients, right or left unilateral modified template was performed. The surgeon classified the resection as complete or incomplete based on surgical findings. In 8 (10.9%) cases the surgeon's opinion was that an incomplete resection was done. Of these cases, in only 1 (1.4%) viable tumor was identified and the follow-up did not show local disease recurrence. An organ resection was necessary in 18 (24.6%) patients, most often a vascular resection (8 cases) or nephrectomy (5 cases). Vascular resections were composed of aorta replacement in 2 cases, vena cava replacement in 2 cases, iliac artery partial resection in 2 cases, aorta and superior mesenteric artery in 1 case and simultaneous aorta and vena cava replacement in 1 case. The characteristics of surgical procedures are demonstrated in Table 3.

**Table 3:** Retroperitoneal Lymph Node Dissection

Characteristic		n	(%)
Template	Bilateral	67	(91.8)
	Extended	4	(5.5)
	Modified Right	1	(1.4)
	Modified Left	1	(1.4)
Nerve sparing	Bilateral	33	(45.2)
	Only left	4	(5.5)
	Only right	5	(6.8)
	No	31	(42.5)
Ureterolysis		7	(9.58)
Operative time (min)		366 (±94)	
EBL (ml)		765 (±1494)	
Packed RBC transfusion (units)		0.96 (±2.3)	
Frozen section		38	(52)
Hospital stay (days)		8.5 (±6.4)	
NGT (days)		2.3 (±1.7)	
Clear liquids (days)		2.9 (±2.5)	

EBL = estimated blood loss, RBC = red blood cells, NGT = nasogastric tube

The most common surgical complications observed were lymphatic leak or lymphocele (10.9%), requiring treatment with percutaneous drainage in 4.1%. Surgical complications are reported in Table 4, according to the Clavien classification.

**Table 4:** Surgical Complications (Clavien Classification)

No complications	45 (61.64%)
I (superficial wound infection, fever)	9 (5.6%)
II (ileus, DVT)	5 (7.0%)
IIIa (symptomatic lymphocele, ureteral leak)	6 (8.4%)
IIIb (laparotomy, incisional hernia)	5 (7.0%)
IVa (acute renal failure, respiratory distress)	2 (2.8%)
IVb (multiple organ failure)	1 (1.4%)
V (death)	0 (0.0%)

The histology from RPLND specimens demonstrated fibrosis or necrosis in 27 (37.0%), teratoma in 30 (41.1%) and viable carcinoma in 16 (21.9%) cases. Among these 16 cases which presented viable cancer, 4 (25%) had metastasis beyond retroperitoneal, 7 (43.8%) showed retroperitoneal masses larger than 5 cm, 8 (50%) presented initial clinical stage IIC or III and 6 (37.5%) required re-induction chemotherapy. However, only 2 (2.7%) of these 16 patients presented high risk disease according to IGCTC at initial assessment. After PC-RPLND, 7 out of 16 patients with viable cancer received additional chemotherapy (adjuvant).

**Figure 2:**
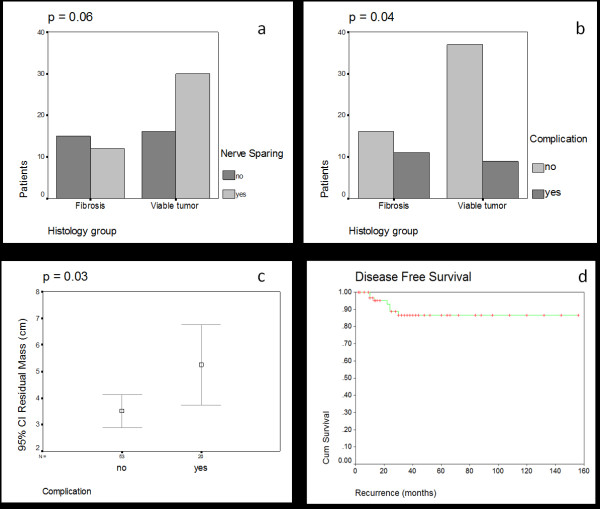
A/B: correlation between PC-RPLND histology and nerve-sparing procedures (A) and surgical complications (B); C: correlation between residual mass size and surgical complications; D: Five-year disease-free survival rate after PC-RPLND.

With respect to predictors of surgical complications, we observed a correlation between the presence of fibrosis with non-nerve sparing surgeries and surgical complications (p < 0.05) (Figure 2a/2b). In other words, in the presence of fibrosis, it was more likely to have a surgical complication or aborted attempt at nerve-sparing. Paradoxically, the patients with tumor (teratoma or carcinoma) were less likely to have a surgical complication or a greater chance at having nerve preservation. None of the variables analyzed correlated with the presence of viable cancer.

The residual tumor size also correlated with complications. The mean size of retroperitoneal disease was 3.2 cm and 2.3 cm, with or without complications, respectively (p = 0.03) (Figure 2c).

After PC-RPLND there were 7 (9.6%) patients with disease recurrence, with only 2 (2.7%) within the previously dissected field; one after a complete bilateral dissection and another one after a right modified dissection. The first patient died due to disseminated neoplasia and the second one remains alive with active disease. Among the 7 patients with recurrences (local or distant), the retroperitoneal pathology was: teratoma (3 cases), embryonal carcinoma (1 case), seminoma, (1 case), adenocarcinoma (1 case) and fibrosis (1 case).

The estimated 5-year overall survival rate after PC-RPLND was 91.2%. The estimated 5-year disease free survival rate was 89.4% (Figure 2d). Median follow-up was 47 months.

## Discussion

Resection of residual tumor after complete serological remission due to first and/or second line chemotherapy in patients with metastatic germ cell tumors is an established staging and therapeutic procedure[9]. However, it is important to continuously evaluate the rates of surgical complications, the presence of residual cancer post-chemotherapy and clinical outcomes.

Defining the patients who actually would benefit the most from this complex surgery is one of the most important issues in the management of this disease. At the moment, there are no reliable methods to select patients with post-chemotherapy residual masses who could be observed without resection [10,11]. Imaging methods are improving yet still fail to determine the favorable group reliably[12]. In this study, we could not identify any factor as a predictor for the presence of viable cancer (risk, size, presence of teratoma, shrinking). Perhaps molecular markers of the primary may assist in the future.

PC-RPLND remains a challenging surgical procedure, especially when large metastases become a fibrotic and adherent to adjacent organs or vital structures[13]. In tertiary centers the overall complications appear comparable to dissections in the primary setting, but there is a tendency of more severe complications in post-chemotherapy patients. Indeed, there is a significantly longer operative time, blood loss, and transfusion rate with PC-RPLND[14,15]. Most common reported complications are ileus and lymph leaks. More rarely, vascular and adjacent organ injuries can occur[16]. Nevertheless, these procedures can be done without excessive risk as demonstrated by several others as well as in this report[17].

The concept of reducing the extent of surgery when fibrosis is detected intra-operatively is appealing. One could consider partial resections or limited templates when only fibrosis is suspected. Actually, this approach was specifically addressed[6] and the authors could predict presence of fibrosis in most cases using intra-operative frozen sections. However, there remains concern regarding this approach since intra-operative frozen section can misclassify disease in approximately 10-15% of cases.

Although intuitively, many believed that severe fibrosis may be linked with complications, our analysis appears to confirm this correlation. Furthermore, although nerve-sparing procedures are feasible in the post-chemotherapy setting[18], in our hands it appears to be more difficult in the presence of fibrosis in residual masses. These two findings represent the dilemma that every surgeon faces when offering PC-RPLND. The patients with higher rates of complications and less likely to undergo nerve-sparing procedures, are exactly those with minimum benefit from the procedure. A reliable predictive tool for viable cancer or teratoma in the post-chemotherapy setting would be most welcome to better select patients and reduce morbidity.

The rational to perform a lymph node dissection in this group of patients is the fact that a significant number of patients will harbor viable teratoma or cancer[2,15]. Although some authors suggest the possibility of performing less extensive templates for selected residual masses after chemotherapy[19], this approach has not been accepted as standard of care and we could not demonstrate a reliable pattern of dissemination to allow safe modified dissections in this setting. The presence of viable cancer post-chemotherapy varies from 4 to 20% among tertiary centers. In our series we observed a slightly higher rate of viable cancer than in the literature. Potential reasons for this observation may include: suboptimal chemotherapy, delays in diagnosis, poor adherence to treatment schedules and complex cases poorly managed in community centres.

Several authors have been emphasizing the relationship between surgical volume and outcomes[20]. Considering the complexity of this procedure and the frequent need for multidisciplinary team assessment, we join those who strongly recommend that the management of patients with metastatic germ cell cancers be restricted to specialized centers.

Finally, we report post-operative complications according to the novel Clavien classification[7] and, as in other areas of urologic surgery, this classification has the potential to standardize the reporting of complications of RPLND. Additional studies using this classification are required in order to validate it in this setting.

## Conclusions

Post-chemotherapy retroperitoneal lymph node dissection is a relatively safe procedure. There remains a significant proportion of patients with teratoma or viable cancer after systemic treatment, justifying surgical resection. The presence of fibrosis and large residual masses is associated with surgical complications and to a non-nerve sparing procedure. There does not appear to be reliable predictors of post-chemotherapy histology of residual masses indicating the continued need for surgical resection in specialized centres.

## Abbreviations

PC-RPLND: Post Chemotherapy Retroperitoneal Lymph Node Dissection; NSGCT: Non-Seminomatous Germ Cell Tumors of the Testis; PHAC : Public Health Agency of Canada; BEP: Bleomycin; Etoposide and Cisplatin; EP: Etoposide and Cisplatin; VIP: Vinblastin; Ifosfamide and Cisplatin; IGCTC: International Germ Cell Consensus Classification

## Competing interests

The authors declare that they have no competing interests.

## Authors' contributions

MAL, AK and SA gathered and compiled data. FB served as pathologist and performed staining and interpretation of slides. AA first conceived of the study and provided valuable input on data evaluation. MAL performed statistical analysis and wrote the manuscript. ST and WK were directly responsible for patient care and data collection. All authors read and approved the final manuscript.
